# Co-Design for People-Centred Care Digital Solutions: A Literature Review

**DOI:** 10.5334/ijic.5573

**Published:** 2021-04-30

**Authors:** M. Ferri Sanz, B. Vallina Acha, M. Ferrando García

**Affiliations:** 1 Kveloce I+D+i (Senior Europa S.L.), ES

**Keywords:** co-design, people-centred care, digital solution, patients, health practitioners, managers

## Abstract

**Introduction:**

The implementation of people-centred care requires strategies that respond to local conditions and contexts, with the participation of local stakeholders in collaborative approaches such as co-design. Within this framework, the authors performed a literature review to identify the most implemented practices in health and social care services for co-designing digital solutions.

**Methods:**

The literature review was conducted following five steps: (i) Definition of the Keywords and their relations; (ii) Definition of the selection criteria; (iii) Search in PubMed; (iv) Selection of papers; and (v) Analysis of the selected papers.

**Results:**

20 papers addressed to co-design health digital solutions with stakeholders were analysed in terms of the activities implemented and participants involved.

**Discussion:**

Previous studies using co-design methods for the deployment of health digital solutions employed a wide range of activities, most of them combining activities and/or mixed target groups.

**Conclusion:**

Co-design is the key to deliver people-centred care as it allows to involve stakeholders in the development of health digital solutions. Implementing one or more of the co-design methods identified in this literature review should be considered to better address the needs and specific projects and target groups.

## Introduction

The **WHO Global Strategy in integrated people-centred health services 2016–2026** calls for a shift in the way health services are funded, managed, and delivered [1]. This strategy highlights the people-centred approach as crucial to the development of health systems that respond to current health challenges, including ageing populations, multi-morbidities, and rising healthcare costs. In this sense, **people-centred care** has been defined as the process of treating the patient as a unique individual [2]. It respects patients and service users as individuals [3] and considers their opinions in decision-making [4]. Consequently, a people-centred approach empowers patients by increasing their role in their own health, giving them information, and providing support, comfort, acceptance, legitimacy, and confidence [5]. According to the WHO^1^ strategy for 2016–2026, the implementation of people-centred care requires strategies that respond to local conditions and contexts, with the participation of local stakeholders and, specially, of disadvantaged populations.

In **Europe**, delivering patient-centred care has become a priority. Indeed, in the 2018 edition of *Health at a Glance* [6] the need for more effective and people-centred health systems, which requires asking patients about their healthcare in a more systematic way, was stressed. In fact, the needs of the patients were moved to the centre of **European public health policy** [7,8]. In this sense, **national** efforts to develop and monitor patient-reported measures have been intensified to respond to the increasing importance of listening and using patients’ voice for developing health systems and improving their quality of care [6].

In this framework, **value-based health care** [9] is a delivery model in which health providers together with citizens/patients aim to reach the best health outcomes for citizens. Thus, the term “value” is derived from measuring health outcomes against the processes and resources needed to achieve those outcomes. Moreover, those outcomes that should be measured and that have “value” are defined and jointly agreed with the person in need of care. Thus, health care providers as well as patients/citizens are engaged in a process of collaboration.

In this context, the **project ValueCare**^1^ aims to deliver efficient outcome-based integrated care to older people facing chronic health conditions in order to improve their quality of life (and that of their relatives) as well as the sustainability of the health and social care systems in Europe. Based on the value-based health approach, ValueCare is developing a robust, secure, and scalable **digital solution** to integrate health and social care and conduct efficient, outcome-based delivery of integrated care solutions for older people.

New technologies have the potential to contribute to more efficient and people-centred care [6]. The European Commission advocates for the digital transformation of health systems to empower citizens to have access to their health data and exchange that data with health professionals. In particular, new technologies can contribute to the transformation of health systems into more integrated and people centred systems needed to respond to the ageing European population. In this sense, digital solutions for health can improve the wellbeing of citizens and change the way health services are delivered if they are designed purposefully and implemented in a cost-effective way. To be effective, they must be designed to meet the needs of both people and health systems [10].

Within the ValueCare project, partners are defining the digital solution following a co-design process with end-users (older people and their families) and service-providers (health and social care professionals, and managers). In this field, **co-design and co-creation** are often confused terms. According to Sanders and Stappers [11], although both terms are activities of collective creativity shared by two or more people, co-creation is a broader term applied from the material to the metaphysical, while co-design is applied across a design process. Thus, co-design appears in a specific phase of the co-creation process. Co-design enables a wide range of people (including professionals and citizens) to bring creative contributions and different perspectives into the discussion. Generally, professionals bring their own expertise and citizens (end-users) share their needs, desires, and requirements.

Within a **health context**, co-design is a method to design better experiences for patients, their caregivers and care professionals [12]. Traditionally, patients and their families were passive recipients of health services, but now considering their inputs into the design and review of services is crucial [13]. Implementing co-design in healthcare is a challenge, especially because of high clinical workloads. However, the benefits of co-design are enormous, in terms of increased staff understanding of patients’ experiences and better experiences for patients [12]. For that reason, the present work **aims** to identify the most implemented practices in health and social care service co-design for digital solutions to guide the co-design process in the ValueCare project. In concrete, this literature review is addressed to map co-design activities implemented in health and social care focused to co-design digital solutions with different target groups (not limiting to any disease or age-group) and with a detailed description of the co-design activities implemented (not guidelines).

## Methods

A literature review was conducted following five steps: (i) Definition of the Keywords and their relations; (ii) Definition of the inclusion criteria; (iii) Search in databases; (iv) Selection of papers; and (v) Analysis of the selected papers. A description of each phase is provided below.

### Definition of the Keywords and their relations

The authors proposed a set of keywords that were presented to the rest of the ValueCare partners in a consortium meeting and later discussed in-depth with the core team working on the co-design guidelines. The final set of key words agreed upon and then used in the review are those included in the following table (***Table 1***).

**Table 1:** Keywords used in the review.

FIELD	CO-DESIGN/CO-CREATION TERM	TARGET GROUP

Health (care) Health and care Social (care) Care services/care pathways value in care Digital health solutions	• Co-design • Co-creation • Contribution	• Patient(s) • Health & social professionals • Health & social managers • Families • Policy makers

Healthcare services	Working with Involving	

Health care services	Accessibility	

Value in care	Expectation	

Sustainability of health- care services	Mutual understanding	

Value in care	Empowerment	


### Definition of the inclusion criteria

Together with the selected keywords, partners agreed to limit the search to papers responding to the following inclusion criteria:

- Scientific papers, as partners agreed to focus only on peer-review papers with evidence-based methodology and results, instead of publications and grey literature;
- Published in the last three years in order to have more updated evidence on co-design activities with the three target groups, namely, adult patients and their families, health and social practitioners, and managers;
- Reports written in English language; and
- Papers with the selected keywords in the title, keywords list or abstract.

### Search in databases

The open database PubMed was selected to carry out the search due to its relevance in the health research field and the introduction in recent years of papers addressed to general care. PubMed comprises more than 30 million citations for biomedical literature from MEDLINE, life science journals and online books.

The first search in PubMed using the key words and inclusion criteria previously defined provided a total of 682 papers and publications.

### Selection of papers

The 682 papers were screened by the authors to eliminate those not related to the research focus or duplicated and include in the analysis only the papers that meet the following criteria:

- reports written in English language;
- covering one of the three target groups defined in the ValueCare project;
- used to create/design a digital health solution/concept for patients/citizens;
- published in the last 3 years; and,
- offering a detailed description of the implemented co-creation activities.

**Figure 1:**
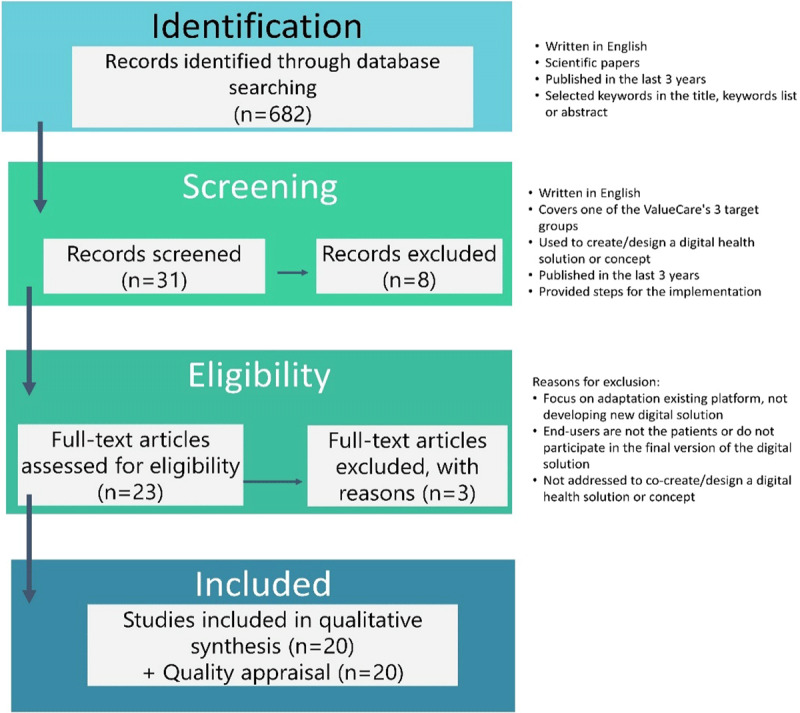
Literature review: adaptation from the PRISMA flow chart.

According to the above selection criteria, 31 papers were selected. Of these, eight papers were eliminated from the study (five papers addressed the target groups of young people and children/parents – those age-groups were eliminated because their high digital skills – and three were not focused on the development of a digital solution), providing at the end a total of 23 papers to be analysed. A final review eliminated 3 more papers that were not on the focus of the ValueCare project (1 was focused to take the most of an existing platform, rather than adapt or develop a new digital solution; other was dedicated to caregivers as end-users of the digital device – not considering patients as end-users; and 1 to community managers views on and experiences with knowledge co-creation in online communities). Therefore, 20 papers meeting the selection criteria were included in the analysis (***Figure 1***). Finally, a quality appraisal was performed to these 20 papers, checking that all of them had been submitted to a peer-review process for external reviewers.

### Analysis of the selected papers

Twenty papers that addressed the co-design of digital solutions with at least one of the ValueCare target groups were analysed in terms of the activities performed and participants involved (***Table 2***). Elements identified include type of activities carried out for each target group, characteristics, organisation, aims, sample size, as well as the evaluation of the co-design processes implemented.

**Table 2:** Studies included in the review.


PAPER TITLE	AUTHOR	YEAR	TARGET PATIENT/POPULATION GROUP	NUMBER OF PARTICIPANTS	CO-DESIGN ACTIVITY	DIGITAL SOLUTION BEING CO-DESIGNED	EVALUATION OF THE CODESIGN PROCESS

Implementing cardiovascular disease prevention guidelines to translate evidence-based medicine and shared decision making into general practice: theory-based intervention development, qualitative piloting and quantitative feasibility	**Bonner et**	**2019**	Health professionals		Small group meetings	Website for GP guidelines, and piloting of a new risk patient calculator/decision aid to help GPs to identify guidelines recommendations for medication and lifestyle change and communicate this to patients.	The co-design process shows how GP and patient feedback can be incorporated into intervention design, but the timeframe required for this process meant that the qualitative analysis was pragmatic rather than formally thematic.

				18	Conference		

				98	Feasibility study		

			Health professionals and patients with cardiovascular disease	10 professionals and 3 patients	Semi-structured interviews		

Digital health technology: factors affecting implementation in nursing homes Digital health technology: factors affecting implementation in nursing homes	**Curtis & Brooks**	**2020**	Manager, nurses, resident and relative	1 manager, 2 nurses, resident and relative	Individual interviews	Digital health technology which includes digital algorithms and digital records	Workshops enabled participating nurses to co-create a three-step process that supported the effective implementation of digital health technology innovations, which have the potential to release staff time, improve quality of care, and have positive effects on staff recruitment and retention. From residents’ point of view, it allows to analyse the level of acceptance of technology in nursing homes.

			Health professionals (nurses)	10	Workshops		

Pilot implementation of co-designed software for co-production in mental health care planning: a qualitative evaluation of staff perspectives	**Farr et al**	**2019**	Health professionals and managers	15 professionals and 5 managers	In-depth interviews	Software forco-production in mental health care planning with interactive touchpoints involving service users.	–

Design and Development of a Context-Aware Knowledge-Based Module for Identifying Relevant Information and Information Gaps in Patients with Type 1 Diabetes Self-Collected Health Data.	**Giordanengo et al**	**2018**	Patients with diabetes	5	Workshop	Prototype for extracting relevant information and documenting information gaps from self-collected health data by patients using a context-aware approach.	–

			Health professionals	4	Workshop		

			Patients and health professionals	9	2 facilitated workshops and a co-design workshop		

Patient-Clinician Co-Design Co-Participation in Design of an App for Rheumatoid Arthritis Management viaTelehealth Yields an App with High Usability and Acceptance	**Grainger et al**	**2017**	Patients and health professionals	9 patients and 11 health professionals	Semi-structured interviews	App for Rheumatoid Arthritis management via Telehealth Yields	–

			Patients with rheumatoid Arthritis	16	Interviews and online survey		

The TiM system: developing a novel telehealth service to improve access to specialist care in motor neurone disease using user-centered design.	**Hobson et al**	**2018**	Patient with Motor Neurone Disease (MND) and public involvement group		Workshops	Telehealth service in MND	Authors strongly recommended user-centred design including all those involved in the receipt and delivery of care whenever a new intervention or service is developed to increase the chances of success.

			Patients with MND and families	1 patient and 1 relative	Semi-structured interviews		

			Patients with MND, families and health professionals	3 patients, 6 carers or ex-carers, and an MND specialist nurse.	Workshops		

			Health professionals	7	Meetings		

			Patient with MND and caregiver		Semi-structured interviews		

			Patients with MND, careers, health professionals		Testing		

Creating Gameful Design in mHealth: A Participatory Co-Design Approach.	**Jessen et al**	**2018**	Patients with chronic conditions	22	Co-design workshops	mHealth self-management app	Participants were both engaged, creative, and voiced a wide range of ideas and requirements; although much of the reported input and ideas were in line with previous research, it provided important contextualization and nuance to these design choices from the users’ perspective.

Design and Development of a Person-Centered Patient Portal Using Participatory Stakeholder Co-Design.	**Kildea et al**	**2019**	Patients with cancer	361	Survey	Person-Centred Patient Portal	As project matured, and more and more stakeholders were engaged, authors noticed an increase in the acceptance by clinical staff of the concept of sharing personal health information with patients

				3 patients	Focus groups		

				5 members of the patient’s committee	Focus groups		

			Health professionals	6	Meetings		

					Presentations		

			Patients with cancer and families	10	End-user testing		

Technology-Enabled Person-Centered Mental Health Services Reform: Strategy for Implementation Science	**LaMonica et al**	**2019**	Health professionals and managers		Survey, semi-structured interviews and workshops	Technology-Enabled Person-Centred Mental Health Services	

			Patients with mental disorders and health professionals		User-testing		

Implementing an Antibiotic Stewardship Information System to Improve Hospital Infection Control: A Co-Design Process	**Maia et al**	**2018**	Health professionals		Survey and interviews	Antibiotic Stewardship Information System	The close collaboration of stakeholders under a participative approach, was the baseline for a successful implementation.

Development of an mHealth platform for HIV Care: Gathering User Perspectives Through Co-Design Workshops and Interviews.	**Marent et al**	**2018**	Patients living with HIV and health professionals	97 patients and 63 health professionals	Co-design workshops	mHealth platform for HIV Care	This process allowed authors to better understand how clinicians and patients were approaching, imagining, and anticipating what the platform could do for HIV care. The co-design approach enabled authors to facilitate early engagement in the mHealth platform, enabling patient and clinician feedback to become embedded in the development process at a pre-prototype phase.

					Semi-structured interviews		

Optimising eHealth tools for older patients: Collaborativeredesign of a hospital website.	**Nguyen et al**	**2018**	Multi-stakeholder related with patients with colorectal cancer	10	Prototype testing	Hospital website	

Co-designing technology with people with dementia and their carers: Exploring user perspectives when co-creating a mobile health application.	**O’Connor**	**2019**	Patients with dementia and families	2 patients and 2 relatives	in-depth interviews	Mobile health application	According to the authors, more participatory methods to create health applications could help patients and carers as they are not often involved in co-producing technology that meets their needs

			Manager and IT expert	1 manager and 1 IT expert	in-depth interviews		

Co-Designing an eHealth Service for the Co-Care of Parkinson Disease: Explorative Study of Values and Challenges.	**Revenäs et al**	**2018**	Patients with Parkinson disease and health professionals	7 patients and 9 health professionals	Co-design workshops	eHealth Service for the Co-Care of Parkinson Disease	Authors concluded that co-design is not mainly about creating new services, but it is about improving current practices to shape better care. Thus, they realised that co-design is only a phase in the cocreation and coproduction of better health care, and its potential can only be realised if the generated ideas are implemented in practice.

Participatory implementation of an antibiotic stewardship programme supported by an innovative surveillance and clinical decision-support system.	**Simões et al**	**2018**	Health professionals		Problem identification (observation) and meetings	Antibiotic stewardship programme supported by an innovative surveillance and clinical decision-support system	

A Collaboration Between Game Developers and Rehabilitation Researchers to Develop a Web-Based App for Persons With Physical Disabilities: Case Study	**Terrill et al**	**2019**	Rehabilitation researchers, software development, people with physical disabilities andclinicians		Design box	Web-Based App for Persons with Physical Disabilities	Authors recognised that engaging stakeholders and end-users early and regularly from initial design ideas to prototype testing is critical. This foster mutual understanding that facilitates coherence within the project while supporting unique professional identities and responsibilities. In fact, it allows synergies in interdisciplinary collaborations that result in better ideas, questions, and solutions than by any one single discipline.

			Multi-stakeholder		User-testing		

A web-based program to improve treatment adherence in patients with type 2 diabetes: Development and study protocol.	**Vluggen**	**2018**	Patients with diabetes, health professionals and IT experts		Program committee	Web-based program to improve treatment adherence	The involvement of relevant stakeholders was an essential element in the development of our computer-tailored program and the subsequent design of the trial.

Conceptual Design and Iterative Development of a mHealth App by Clinicians, Patients and Their Families	**Woods et al**	**2018**	Health professionals		Prototype testing	mHealth App	Using participatory design processes allowed for the inclusion of diverse perspectives from different stakeholders into the product’s features and functions. According to the authors, accurate, evidence-based and validated mHealth apps, if designed with a balance of consumer and provider input, can be safely used at home.

Design Thinking for mHealth Application Co-Design to Support Heart Failure Self-Management.	**Woods et al**	**2017**	Patients with heart failure and families		Ethnographic interviews	mHealthApplication	Authors concluded that the systematic design process provides a robust evidence-base for their speciality in health technology design for the advancement of patient-centred care.

			Patients with heart failure	12	Interviews		

Co-Design of a Mobile Health App for Heart Failure: Perspectives from the Team	**Woods et al**	**2019**	Health professionals, patients with heart failure and family	11	Interviews	Mobile Health App	Analysis of stakeholders’ accounts of the co-design process has enabled a deeper understanding of the strengths and weaknesses in operationalising co-design. As conclusions, authors stated that co-design can be achieved with a sincere partnership between staff and consumers. The findings suggested that managing stakeholders throughout the design is key to the project’s success.

			Multi-stakeholder	7 health professionals, 7 patients and 4 caregivers	Design workshops		

					Prototype		


## Results

The results of the analysis are presented below by the target group addressed (see ***Table 3***).

**Table 3:** Overview of the literature review results by target group.


TARGET	CO-DESIGN ACTIVITIES	NUMBER OF PAPERS

Patients (and their caregivers)	Surveys, interviews, focus groups, workshops and testing sessions	10 papers

Health and social practitioners	Interviews, meetings, presentations, observation, workshops, survey and user-testing sessions	11 papers

Managers	Interviews	2 papers

Multi-stakeholder target groups	Interviews, workshops, feedback sessions, meetings, design box session, presentations	9 papers


### Patients

Identified studies involved patients, some of them also their caregivers, using surveys, interviews, focus groups, workshops, and testing sessions in the co-design of different people-centred digital solutions.

**Surveys:** Patients with cancer [14] and with rheumatoid arthritis [15] were involved in the design of people-centred health apps. Patients with cancer were surveyed after the development of the app to confirm their preferences and verify that the software design was aligned with their preferences. In the case of patients with rheumatoid arthritis, they tested the app prototype for one month and provided their feedback in a dedicated survey. Surveys were conducted in person (in waiting rooms of the cancer centre) and online, including the System Usability Scale and free text feedback. In relation to the sample size, these studies achieved a convenience-sample of 361 patients [14] and 16 [15] who participated voluntarily in the survey.

**Interviews:** Included studies used **semi-structured interviews** [15,16,17], **in-depth interviews** [18] and **ethnographic interviews** [19].

Patients with rheumatoid arthritis [15] and with heart failure [19] were interviewed twice along the co-design process. The aim of the first interview was to explore technology use, assess digital literacy, and to understand how these patients self-manage their health in daily life. The second interview was performed post app download [15] and after interacting with the app for 14 days [19]. Patients living with HIV [16] were also interviewed to facilitate the co-design process of a mHealth platform to be integrated into clinical care pathways. HIV patients were asked about topics similar to the interviews performed by Grainger et al [15] and Woods et al [19] with rheumatoid arthritis and heart failure patients respectively: HIV patients were asked about their experiences of living with HIV, functionalities for the mHealth solution, and barriers/concerns. Patients with cardiovascular disease were interviewed to collect data from a functional website prototype, once it included inputs from interviews conducted with general practitioners (GPs) [17]. Residents of nursing homes and their relatives were interviewed to explore their understanding of digital health technology and their experiences using it in healthcare settings [20]. People with dementia and their careers were interviewed to co-create a mobile health application [18].

Interviews were continued until saturation was achieved: 9 patients with rheumatoid arthritis were interviewed [15] and the same numbers were achieved in the case of patients with cardiovascular disease [17]. Woods et al [19] involved 12 patients, Marent et al [16] performed 20 interviews to validate the tool, Curtis and Brooks [20] interviewed 4 residents in nursing homes and their relatives in each nursing home participating in the study (that is, a total of 20 interviews), and O’Connor [18] interviewed 2 people with dementia and 2 careers.

**Focus groups:** Patients of a care centre participated in one focus group implemented to co-design and pilot test a person-centred patient portal smartphone app [14]. In this study, the focus group was complemented with another focus group with members of the patients’ committee of the same cancer centre. The aims of those focus groups were the following:

- the focus group with patients (3 participants) tested the app prototype in terms of features and usability. The radiation therapy team helped identify patients who had finished their treatment to be engaged in the focus group. A total of ten patients were identified but only three participated in the focus group. Those not attending to the focus group indicated they were unavailable at the time and date chosen. The app was presented during the focus group and participants were observed while using it. Later on, moderators went through each feature asking for patients’ feedback.
- the focus group with the patients’ committee (5 members) rehearsed the registration process and anticipated initial real-world problems.

**Workshops:** People living with HIV participated in a total of ten workshops (including three which were mixed workshops with clinicians) at the offices of community partners, hotels or in the clinic, depending on what was appropriate [16]. The authors combined the workshops with semi-structured interviews with the objective of facilitating the co-design process of a mHealth platform to be integrated into clinical care pathways. In the same line, 22 adults with chronic conditions participated in three sets of two consecutive co-design workshops using frames, scenarios, prototypes, and sticky notes [21]. The aim of those workshops was to explore user needs, preferences, and ideas to implement playful designs in a health self-management app.

**User testing sessions:** Users of a mental health service participated in baseline and follow-up user-testing sessions to gather ongoing feedback on a technology-enabled solution for mental health services reform for continuous design and development of the tool [22].

### Healthcare practitioners

Included studies under review involved healthcare practitioners in the co-design process of digital solutions through interviews, meetings, presentations, observation, workshops, surveys, and user-testing sessions:

**Interviews:** A number of studies used **semi-structured interviews** [15,16,17,22,23] and **in-depth telephone interviews** [24].

Semi-structured interviews were conducted with:

- 11 health care professionals to explore technology use, app functionality, barriers and facilitators to app use, and potential impacts of app implementation on rheumatoid arthritis service provision and experience [15];
- clinicians to (1) elicit experiences of working in HIV care, (2) identify mHealth functionalities that are considered useful for HIV care, and (3) identify potential benefits as well as concerns about mHealth [16];
- 10 GPs to test a preliminary website to support GPs in cardiovascular disease prevention, 7 of them while using the preliminary website and 3 of them once the changes were incorporated according to the feedback gathered in the first interviews [17];
- 2 nurses in four nursing homes to explore their understanding of digital health technology and their experiences using it in healthcare settings [20]; and
- health professionals during a 12-month process to optimise technology-enabled solutions for mental health services reform with the aim of assessing digital readiness and competence, clinician behaviours, attitudes, skills, and knowledge (baseline and follow-up interviews) and retrospective review of technology and social return of investment (follow-up interviews) [22].

In-depth telephone interviews were used to collect data from 15 health professionals working in mental health, about their views and experiences with an electronic care pathway tool, acceptability, and feasibility of using this tool, impact on working practice and ability to co-produce care plans, and suggested improvements [24].

Interviews were also used by Maia et al [25] to co-design and implement an antibiotic stewardship information system to improve hospital infection control via an effective surveillance and decision support system adapted to the local socio-cultural context. With the same aim, Simões et al [23] conducted semi-structured interviews supported by a pre-elaborated questionnaire about the usefulness of the tool to all healthcare workers involved in antibiotic monitoring and prescription processes (infection control team, physicians, pharmacy, and microbiology laboratory staff).

**Meetings:** GPs were involved in two small group meetings to co-develop the website content for GPs’ guidelines and a new risk calculator/decision aid in cardiovascular disease prevention [16]. Simões et al [23] observed healthcare workers for four hours during the morning (the period of the day with more work related to antibiotic prescription practices). Then, meetings were conducted to characterise and understand their workload related to these practices.

**Presentations:** Continuous staff input was gathered by Kildea et al [14] with presentations of the planned features and functionality of an app to various staff groups, from on-the-floor care providers to the institution board of directors to ensure awareness at all levels, to obtain staff feedback and address their concerns, and to seek support to continue developing the person-centred patient portal smartphone app. Those presentations were combined with other co-design activities to develop the person-centred app for cancer patients as survey to patients, focus groups with patients, and end-users testing. Bonner et al [17] organised a conference for GPs via a presentation and question/answer session and displayed a tablet in an exhibition room to pilot a website prototype to support cardiovascular disease prevention.

**Workshops:** Marent et al [16] conducted seven workshops with clinicians (three of which were mixed workshops with people living with HIV). The authors combined the workshops with semi-structured interviews with the objective of facilitating the co-design process of a mHealth platform to be integrated into clinical care pathways. LaMonica et al [22] organised baseline workshops with service staff working on mental health (health professionals, administrators, and service managers) and service consumers to further assess digital readiness and competence, change impact, quality, usability, and acceptability in a group setting, together with three more follow-up workshops to include retrospective review of technology, and social return on investment. This activity complemented the survey and interviews also conducted in this study. Curtis and Brooks [20] implemented two co-creation workshops with ten nurses who previously participated in interviews. These workshops aimed to design a nurse-led process to support implementation of a digital health technology innovation in nursing homes.

**Survey:** A survey was implemented (pre and post) to perform a feasibility study to assess the acceptability, demand, and potential efficacy of the final version of a website to support GPs dealing with cardiovascular disease [17]. A 10-minute baseline survey was completed by 123 GPs, followed by a 4-minute post-evaluation survey sent one month after completion of the baseline survey, and completed by 98 GPs. Similarly, LaMonica et al [22] conducted a baseline survey to assess digital readiness, change impact, quality, usability and acceptability to health professionals of a technology-enabled person-centred mental health services reform tool. This was combined with 4 follow-up surveys after three, six, nine and twelve months from the baseline survey to assess changes to previous questions and the social return of the investment. A survey was also used by Maia et al [25] to co-design and implement an antibiotic stewardship information system to improve hospital infection control.

**User testing sessions:** LaMonica et al [22] implemented baseline and follow-up user-testing sessions to gather ongoing feedback on a technology-enabled solution for mental health services reform for continuous design and development of the tool.

### Managers

Managers have been involved in co-design through **in-depth telephone interviews**. Farr et al [24] interviewed five managers to gather information about strategic perspectives affecting the adoption of new IT innovation, implementation, and organisation in mental health care planning. A manager in four nursing homes was also interviewed to explore his/her understanding of digital health technology and experiences using it in healthcare settings [20].

### Co-design activities with mixed target groups

Some authors implemented co-design processes involving different target groups in the same activity:

**Workshops:** Woods et al [19,26] created a co-design team composed of patients (adults with heart failure), careers, clinicians, an app developer, and a research team. This group was involved in design thinking process that considered, among other activities, a two-hour collaborative design workshop for idea generation using creative thinking activities such as Idea Matrix, and a second convergent thinking approach workshop to review and discuss the outcomes derived from the first workshop and select the best idea. In the same line, two participative design workshops led by two nurses and conducted with six clinicians and a patient were employed by Woods et al [27].

Revenäs et al [28] conducted four half-day co-design workshops with seven people with Parkinson disease and nine health care professionals with the aim of designing an eHealth service for co-care Parkinson disease. According to the author [28], the term co-care specifically stresses the combination of health care professionals’ and patients’ resources, supported by appropriate (digital) tools for information exchange, to achieve the best possible health outcomes for patients. The first three workshops were used to capture needs and generate ideas for the eHealth service, and the fourth workshop was focused on demonstrating the prototype of the mobile app.

Moreover, Hobson et al [29] implemented two workshops with three patients with motor neurone disease, six carers or ex-carers, and a specialist nurse. The workshops were facilitated by a clinician, a specialist in user-centred design and a telehealth user experience designer and used practical dynamics (puzzles, patient journey mapping exercise, personas). Feedback was requested in writing at the end of the session. Those activities were combined with consultation with health professionals and a final testing with users. Giordanengo et al [30] implemented two facilitated workshops with the participation of the author, one patient with diabetes and one clinician with the aim of designing a knowledge-based module.

**Multidisciplinary consensus meetings/sessions:** Nguyen et al [31] implemented a co-design process with patients and professional stakeholders (researchers, physicians, nurses, managers, policymakers, and website designers) to redesign an existing hospital website with the objective of making it more user-friendly for older patients with colorectal cancer. The co-design process was performed in three phases starting from the content and design evaluation to the prototype testing and assessment. In a similar way, a co-design session involving five patients with diabetes, four clinicians (two endocrinologists and two diabetes nurses) was organised by Giordanengo et al [30] structured into three sub-sessions: patients only, clinicians only, and all participants together. Vluggen et al [32] created a programme committee to foster the co-creation process of a web-based programme to improve treatment recommendation adherence in patients with type 2 diabetes. The committee was composed of practice nurses, diabetes nurses, a dietician, an internist, a general practitioner, health scientists, eHealth experts and patients with type 2 diabetes. They met three times during the 18-month programme development.

**Design box:** A design box to discuss the needs and key elements of the app was used by rehabilitation researchers (three clinical researchers and a social scientist), software developers, people with physical disabilities and clinicians to co-design a web-based app for persons with physical disabilities. The design box is a participatory, inductive design methodology, that foster the collaboration between users (in this case, rehabilitation researchers) as designers (in this case, software developers). The box allows them to discuss about four topics (each of them in one of the sides of the box): (i) audience, that is, anyone that could say no to the product (i.e. clinical professionals, project founders); (ii) technology development; (iii) aesthetic, those elements should focus on how the end-user will feel when using the software; and (iv) problem statement, that sums up the problems the research team is trying to solve. Then, the prototype was developed and later tested by users to identify errors and gather feedback on usability and accessibility [33].

**Interviews:** Woods et al [26] implemented interviews with patients, caregivers, and clinicians to capture experience data and needs using creative representations (journey map, stakeholder map, and patient personas). In total, three patients, one caregiver and nine health professionals (a cardiac nurse consultant, a cardiologist, a physiotherapist, a dietitian, a pharmacist and two heart failure nurse practitioners) were interviewed. Hobson et al [29] also implemented a semi-structured interview with a patient and caregiver who lived at a distance from the location of the meetings to complement the other co-design activities implemented in their study (presentations and workshops).

**Presentations:** Hobson et al [29] presented early ideas on a novel telehealth service to improve access to specialist care in motor neurone disease at three meetings with patients and members of the public, and two local associations in the field of the target disease.

## Discussion

This review showed a variety of tools used to co-design digital health solutions: surveys, focus groups, interviews, workshops, meetings, presentations, and observation. Interviews, used by 11 studies [15,16,17,18,19,22,23,24,26,29], were the most widely used tool for the co-design of digital solutions in the health field. Workshops were used by nine studies [16,19,20,21,22,26,28,29,30] and meetings by six studies [17,19,23,29,31,32]. Focus groups were used only by one study [14] although the description of the workshops in the papers reviewed is quite similar to the focus groups. Surveys were also less used, with only four studies using this tool to collect information for the development of digital solutions [14,15,22,25].

Most of the studies reviewed combined tools and involved different target groups in the co-design activities (see ***Table 4*** below). Some of them were addressed to a unique target group (patient or health professional) but most authors combined activities and/or mixed target groups. Most of the studies involved patients (and their families) and health professionals in the co-design process (a total of nine studies) and combined at least two types of co-design activities (six studies). Four studies involved a multi-stakeholder group with health professionals, researchers, policy makers, patients, managers, using multidisciplinary consensus meetings [31], surveys, interviews, and user-testing sessions [21], workshops [19,22] and a design box [33]. Two studies involved only one target group and one activity type. This is the case of patients (and their caregivers) with workshops [21] and interviews [18]. Similarly, two studies involved only health professionals, but combined co-design activities: interviews and surveys [25] and observation, meetings, and interviews [23].

**Table 4:** Overview of co-design activities and target groups involved in the included studies.


PATIENTS (AND CAREGIVERS)			PATIENTS AND HEALTH PROFESSIONALS	

Workshops		3 sets of 2 consecutive co-design workshops [21]	Workshops	Mixed workshops [28,29]
Interviews		In-depth interviews with people with dementia and their caregivers [18]	Meetings	3 meetings during 18-months process of programme developed [32]

			Survey and interviews	Survey and semi-structured interviews with patients and health professionals [15]

**Only health professionals**			Interviews and workshops	Semi-structured interviews with patients, and combination of workshops with patients, health professionals and mixed [16]

Interviews and survey [25]				In-person survey in parallel to the software design to confirm patient preferences and verify that the software was being developing in this line, focus groups for the app prototype testing with patients, and presentations to health professionals along the app development [14].
Observation, meetings and interviews		Semi-structured interviews, 4-hours observation and meetings [23]	Survey, focus groups and presentations	

**Health professionals and managers**			Interviews, workshops, and meetings	Interviews with patients and families not able to attend to the workshops, workshops with patients and professionals, and meetings with health professionals [29]. Interviews with patients, caregivers and clinicians to capture experience data and needs using creative representations, plus 2-hours collaborative design workshop for the idea generation and feedback sessions to test prototypes [26] Interviews to patients and health professionals plus meetings and presentations to health professionals [17]
Interviews	In-depth interviews [24]			

**Patients, health professionals and managers**				

Interviews and workshops		Interviews with residents in nursing homes, nurses, and managers, and 2 workshops with nurses [20]	Interviews, meetings, and presentations	

**Patients and professionals’ stakeholders (health professionals, researchers, managers, policy makers)**				

Meetings		Multidisciplinary consensus meetings [31]		

Survey, semi-structured interviews, workshops, user-testing		Ongoing feedback from service staff is collected via online surveys, semi-structured interviews, and workshops to evaluate and monitor the impact of embedding the technology solution. Staff and consumers feedback about existing and newly functionalities are collected with quarterly user testing sessions. Interviews with patients, nurses, and managers of nursing homes and 2 workshops with nurses [22]		

workshops		Series of workshops from the idea generation to the consensus regarding the features and functions of the wireframes plus Ethnographic interviews with patients [19]		

Design box		The needs and key elements of the app were discussed. Then, the prototype was developed and later tested by users to identify errors and gather feedback on usability and accessibility [33]		


An overview of the activities used in the included studies is presented in the following table (***Table 4***):

## Conclusion

The implementation of people-centred care requires strategies that respond to local conditions and contexts and that involve local stakeholders [1]. European member states have increased their efforts to listen patients’ voices in the delivery of health care and to improve its quality [6]. In this context, digital innovations can greatly contribute to achieve more efficient and people-centred care [6] if they are designed to meet the needs of people as well as health systems [10]. Co-design was revealed as a key method to deliver people-centred care as it allows the involvement of stakeholders (patients, health professionals, managers, policy makers, and others) in the development of the health digital solution, and responds to their needs and requirements as a way to increase the usefulness and sustainability of the digital innovation.

This review provides evidence that a combination of co-design tools and stakeholder groups (as detailed in ***Table 4***), mainly those directly affected by the digital tool (patients and health professionals) are the most common approaches to implementing co-design in health systems for the deployment of digital solutions. Among the papers reviewed, the **main implemented co-design activities were interviews and workshops** followed by **meetings** and **surveys**. Interviews were used with patients to explore technology use [15] and gather feedback about prototypes [16]; with families of residents in nursing homes [20]; with both target groups together (families and patients) to co-design an app [18]; and with health care professionals and managers by phone to co-produce a software with interactive touch points between health professionals and end-users [24] or in-person to design a mobile health application [18]. Workshops were used with patients [21] to co-design a mHealth self-management app, and health and social professionals [22] to develop a technology-enabled person-centred service.

In the analysed papers, authors stated that the stakeholders’ participative approach was the baseline for a successful implementation of the digital health devices [25,32]. Participative approaches allow for a better understanding of the professionals and patients’ needs and requirements [18,27,26] facilitating at the same time coherence within the project [14,33], and end-users engagement in the digital device use from early stages [16]. Consequently, co-design is recognised as a tool to increase the chances of success of the digital device being developed [30]. For that reason, and based on the literature review, authors provide the following recommendations to researchers willing to implement co-design activities to develop digital health and social solutions involving patients, health professionals and managers as a way to deliver people centred care: (i) involve them from the beginning of the digital solution idea; (ii) combine co-design activities (i.e. workshops with interviews); (ii) combine sessions addressed to only one target group with mixed sessions involving different target groups (i.e. patients and health professionals); (iv) and, if possible, add a session to test the device prototype with real users.

The limited information provided by some of the reviewed papers in relation to the number of participants and the process of implementing the co-design activities is a constraint of this study. Further research may explore middle-range theory building through meta-synthesis of the 20 studies to extract the successful aspects of the co-design activities. This would deliver practical and useful recommendations to new researchers seeking to implement co-design activities to develop digital health solutions involving patients, health professionals and managers as a way to deliver people-centred care. Another limitation of the study lies in the use of PubMed as the unique database for the literature review. In this sense, other databases with a traditional social perspective could have provided more insights about the use of co-design activities in social care.

## Footnotes

Project ValueCare – Value-based methodology for integrated care supported by ICT – co-funded by the European Union’s Horizon 2020 research and innovation programme under grant agreement No 875215. Project website: *https://projectvaluecare.eu/*.

## References

[1] World Health Organisation [Internet]. WHO global strategy on integrated people-centred health services 2016–2026 services, 2015. Available from: https://www.who.int/servicedeliverysafety/areas/people-centred-care/global-strategy/en/.

[2] Redman RW. Patient-centered care: an unattainable ideal? Res Theory for Nurs Pract. 2004; 18(1): 11–14. Available from: https://pubmed.ncbi.nlm.nih.gov/15083659/. DOI: 10.1891/rtnp.18.1.11.28057 15083659

[3] Binnie A, Titchen A. Freedom to practise: the development of patient-centred nursing. London: Butterworth Heinemann; 1999.

[4] Shaller D. Patient-centered care: what does it take? Picker Institute, Oxford and The Commonwealth Fund. 2007. [cited 2020 April 16]. Available from: https://www.issuelab.org/resources/10548/10548.pdf.

[5] Fulford KWM, Ersser S, Hope T. Essential practice in Patient-centred care. UK: Blackwell Science Ltd; 1996.

[6] OECD/EU. Health at a Glance: Europe 2018: State of Health in the EU Cycle. Paris: OECD Publishing; 2018. DOI: 10.1787/health_glance_eur-2018-en

[7] Byrne, D. Patient centred health policy in Europe. European Commissioner for Health and Consumer protection. European Federation of Pharmaceutical Industries (EFPIA). Dublin: Public Conference; 2004.

[8] European Union. State of Health in EU. Companion report 2017. Luxemburg publications office of European Union, 2017. ISBN 978-92-79-73492-2.

[9] NEJM Catalyst. What is value-based healthcare? Innovations in Care Delivery [Internet]. Date of publication: 2017, 1 1 [16 April 2019] Available from: https://catalyst.nejm.org/what-is-value-based-healthcare/.

[10] European Commission. Communication from the Commission to the European parliament, the council, the European Economic and Social Committee and the Committee of regions on enabling the digital transformation of health and care in the Digital Single Market; empowering citizens and building a healthier society. 2018. COM (2018) 233 final. [cited 2020 April 16]. Available from: https://ec.europa.eu/digital-single-market/en/news/communication-enabling-digital-transformation-health-and-care-digital-single-market-empowering.

[11] Sanders EBN, Stappers PJ. Co-creation and the new landscapes of design. International Journal of CoCreation in Design and the Arts. 2008; 4(1): 5–18. DOI: 10.1080/15710880701875068

[12] NHS Institute for Innovation and Improvement. The ebd approach – Guide and Tools. Coventry: NHS Institute for Innovation and Improvement; 2019.

[13] Bate P, Robert G. Bringing User Experience to Healthcare Improvement. Oxon: Radcliffe Publishing Ltd; 2007.

[14] Kildea J, Battista J, Cabral B, Hendren L, Herrera D, Hijal T, Joseph A. Design and Development of a Person-Centered Patient Portal Using Participatory Stakeholder Co-Design. J Med Internet Res [Internet]. 2019; 21(2). [cited 2020 April 20]. Available from: https://pubmed.ncbi.nlm.nih.gov/30741643/. DOI: 10.2196/preprints.11371 PMC6388099 30741643

[15] Grainger R, Townsley H, Langlotz T, Taylor W. Patient-Clinician Co-Design Co-Participation in Design of an App for Rheumatoid Arthritis Management via Telehealth Yields an App with High Usability and Acceptance. Stud Health Technol Inform. [Internet]. 2017; 245: 1223. [cited 2020 April 20]. Available from: https://pubmed.ncbi.nlm.nih.gov//29295310/. 29295310

[16] Marent B, Henwood F, Darking M. EmERGE Consortium. Development of an mHealth platform for HIV Care: Gathering User Perspectives Through Co-Design Workshops and Interviews. JMIR Mhealth Uhealth [Internet]. 2018; 6(10): e184. [cited 2020 April 20]. Available from: https://pubmed.ncbi.nlm.nih.gov/30339132/. DOI: 10.2196/preprints.9856 30339132 PMC6231792

[17] Bonner C, Fajardo MA, Doust J, McCaffery K, Trevena L. Implementing cardiovascular disease prevention guidelines to translate evidence-based medicine and shared decision making into general practice: theory-based intervention development, qualitative piloting and quantitative feasibility. Implement Sci. [Internet] 2019; 14(1): 86. Published 2019 Aug 30. DOI: 10.1186/s13012-019-0927-x 31466526 PMC6716813

[18] O’Connor S. Co-designing technology with people with dementia and their carers: Exploring user perspectives when co-creating a mobile health application. Int J Older People Nurs [Internet]; 2019; e12288. DOI: 10.1111/opn.12288 31837096

[19] Woods L, Cummings E, Duff J, Walker K. Design Thinking for mHealth Application Co-Design to Support Heart Failure Self-Management. Stud Health Technol Inform [Internet]; 2017; 241: 97–102. [cited 2020 April 20]. Available from: https://pubmed.ncbi.nlm.nih.gov/28809190/. 28809190

[20] Curtis K, Brooks S. Digital health technology: factors affecting implementation in nursing homes. Nurs Older People [Internet]. 2020; 32(2): 14–21 [cited 2020 April 20]. Available from: https://pubmed.ncbi.nlm.nih.gov/32159302/. DOI: 10.7748/nop.2020.e1236 32159302

[21] Jessen S, Mirkovic J, Ruland CM. Creating Gameful Design in mHealth: A Participatory Co-Design Approach. JMIR Mhealth Uhealth [Internet] 2018; 6(12): e11579. Available from: https://pubmed.ncbi.nlm.nih.gov/30552080/. DOI: 10.2196/11579 30552080 PMC6315237

[22] LaMonica HM, Davenport TA, Braunstein K, et al. Technology-Enabled Person-Centered Mental Health Services Reform: Strategy for Implementation Science. JMIR Ment Health [Internet]. 2019; 6(9): e14719. Available from: https://pubmed.ncbi.nlm.nih.gov/31538938/. DOI: 10.2196/14719 31538938 PMC6786853

[23] Simões AS, Maia MR, Gregório J, et al. Participatory implementation of an antibiotic stewardship programme supported by an innovative surveillance and clinical decision-support system. J Hosp Infect [Internet]. 2018; 100(3): 257–264. [cited 2020 April 20]. Available from: https://pubmed.ncbi.nlm.nih.gov/30071264/. DOI: 10.1016/j.jhin.2018.07.034 30071264

[24] Farr M, Pithara C, Sullivan S, et al. Pilot implementation of co-designed software for co-production in mental health care planning: a qualitative evaluation of staff perspectives. J Ment Health [Internet]. 2019; 28(5): 495–504. [cited 2020 April 20]. Available from: https://pubmed.ncbi.nlm.nih.gov/31240971/. DOI: 10.1080/09638237.2019.1608925 31240971

[25] Maia MR, Simões A, Lapão LV. Implementing an Antibiotic Stewardship Information System to Improve Hospital Infection Control: A Co-Design Process. Stud Health Technol Inform [Internet]. 2018; 247: 56–60. [cited 2020 April 20]. Available from: https://pubmed.ncbi.nlm.nih.gov/29677922/. 29677922

[26] Woods L, Roehrer E, Duff J, Walker K, Cummings E. Co-design of a mobile health app for heart failure: perspectives from the team. Studies in Health Technology and Informatics. 2019; 266: 183–188. [cited 2020 April 20]. Available from: https://pubmed.ncbi.nlm.nih.gov/31397321/. 31397321 10.3233/SHTI190792

[27] Woods L, Cummings E, Duff J, Walker K. Conceptual Design and Iterative Development of a mHealth App by Clinicians, Patients and Their Families. Stud Health Technol Inform [Internet]. 2018; 252: 170–175. [cited 2020 April 20]. Available from: https://pubmed.ncbi.nlm.nih.gov/30040701/. 30040701

[28] Revenäs Å, Hvitfeldt Forsberg H, Granström E, Wannheden C. Co-Designing an eHealth Service for the Co-Care of Parkinson Disease: Explorative Study of Values and Challenges. JMIR Res Protoc [Internet]. 2018; 7(10): e11278. [cited 2020 April 20]. Available from: https://pubmed.ncbi.nlm.nih.gov/30377143/. DOI: 10.2196/11278 30377143 PMC6234336

[29] Hobson EV, Baird WO, Partridge R, et al. The TiM system: developing a novel telehealth service to improve access to specialist care in motor neurone disease using user-centered design. Amyotroph Lateral Scler Frontotemporal Degener [Internet]. 2018; 19(5–6): 351–361. [cited 2020 April 20]. Available from: http://eprints.whiterose.ac.uk/128397/1/The%20TiM%20system%20developing%20a%20novel%20telehealth%20service%20to%20improve%20access%20to%20specialist%20care%20in%20motor%20neurone%20disease%20using%20user%20centered%20design.pdf. DOI: 10.1080/21678421.2018.1440408 29451026

[30] Giordanengo A, Øzturk P, Hansen AH, Årsand E, Grøttland A, Hartvigsen G. Design and Development of a Context-Aware Knowledge-Based Module for Identifying Relevant Information and Information Gaps in Patients With Type 1 Diabetes Self-Collected Health Data. JMIR Diabetes. [Internet] 2018; 3(3): e10431. [cited 2020 April 20]. Available from: https://www.ncbi.nlm.nih.gov/pmc/articles/PMC6238884/. DOI: 10.2196/preprints.10431 30291097 PMC6238884

[31] Nguyen MH, Bol N, van Weert JCM, et al. Optimising eHealth tools for older patients: Collaborative redesign of a hospital website. Eur J Cancer Care (Engl). [Internet]. 2019; 28(1): e12882. [cited 2020 April 20]. Available from: https://pubmed.ncbi.nlm.nih.gov/30015998/. DOI: 10.1111/ecc.12882 30015998 PMC6588263

[32] Vluggen S, Hoving C, Schaper NC, de Vries H. A web-based program to improve treatment adherence in patients with type 2 diabetes: Development and study protocol. Contemporary Clinical Trials [Internet] 2018; 74: 38–45. ISSN 1551-7144. [cited 2020 April 20]. Available from; https://pubmed.ncbi.nlm.nih.gov/30290275/. DOI: 10.1016/j.cct.2018.10.002 30290275

[33] Terrill AL, MacKenzie JJ, Reblin M, Einerson J, Ferraro J, Altizer R. A Collaboration Between Game Developers and Rehabilitation Researchers to Develop a Web-Based App for Persons With Physical Disabilities: Case Study. JMIR Rehabil Assist Technol [Internet]. 2019; 6(2): e13511. [cited 2020 April 20]. Available from: https://pubmed.ncbi.nlm.nih.gov/31573927/. DOI: 10.2196/13511 31573927 PMC6789424

